# Polyphenols from Bee Pollen: Structure, Absorption, Metabolism and Biological Activity

**DOI:** 10.3390/molecules201219800

**Published:** 2015-12-04

**Authors:** Anna Rzepecka-Stojko, Jerzy Stojko, Anna Kurek-Górecka, Michał Górecki, Agata Kabała-Dzik, Robert Kubina, Aleksandra Moździerz, Ewa Buszman

**Affiliations:** 1 Department of Pharmaceutical Chemistry, School of Pharmacy with the Division of Laboratory Medicine, Medical University of Silesia, Jagiellońska 4, Sosnowiec 41-200, Poland; ebuszman@sum.edu.pl; 2 Department of Hygiene, Bioanalysis and Environmental Studies, School of Pharmacy with the Division of Laboratory Medicine, Medical University of Silesia, Kasztanowa 3A, Sosnowiec 41-200, Poland; jstojko@sum.edu.pl (J.S.); amozdzierz@sum.edu.pl (A.M.); 3 Silesian Medical College in Katowice, Mickiewicza 29, Katowice 40-085, Poland; anna.kurek_gorecka@swsm.pl; 4 Department of Drug Technology, School of Pharmacy with the Division of Laboratory Medicine, Medical University of Silesia, Jedności 8, Sosnowiec 41-200, Poland; mgorecki@sum.edu.pl; 5 Department of Pathology, School of Pharmacy with the Division of Laboratory Medicine in Sosnowiec, Medical University of Silesia in Katowice, Ostrogórska 30, 41-200 Sosnowiec, Poland; adzik@sum.edu.pl (A.K.-D.); rkubina@sum.edu.pl (R.K.)

**Keywords:** bee pollen, antioxidants, polyphenols, natural product

## Abstract

Bee pollen constitutes a natural source of antioxidants such as phenolic acids and flavonoids, which are responsible for its biological activity. Research has indicated the correlation between dietary polyphenols and cardioprotective, hepatoprotective, anti-inflammatory, antibacterial, anticancerogenic, immunostimulating, antianaemic effects, as well as their beneficial influence on osseous tissue. The beneficial effects of bee pollen on health result from the presence of phenolic acids and flavonoids which possess anti-inflammatory properties, phytosterol and linolenic acid which play an anticancerogenic role, and polysaccharides which stimulate immunological activity. Polyphenols are absorbed in the alimentary tract, metabolised by CYP450 enzymes, and excreted with urine and faeces. Flavonoids and phenolic acids are characterised by high antioxidative potential, which is closely related to their chemical structure. The high antioxidant potential of phenolic acids is due to the presence and location of hydroxyl groups, a carboxyl group in the immediate vicinity of *ortho*-diphenolic substituents, and the ethylene group between the phenyl ring and the carboxyl group. As regards flavonoids, essential structural elements are hydroxyl groups at the C5 and C7 positions in the A ring, and at the C3′ and C4′ positions in the B ring, and a hydroxyl group at the C3 position in the C ring. Furthermore, both, the double bond between C2 and C3, and a ketone group at the C4 position in the C ring enhance the antioxidative potential of these compounds. Polyphenols have an ideal chemical structure for scavenging free radicals and for creating chelates with metal ions, which makes them effective antioxidants *in vivo*.

## 1. Introduction

In recent years, there has been an increased interest in the addition of bioactive ingredients from plants, such as polyphenols, to food. A correctly composed diet enriched in polyphenols may counteract the development of many diseases. Polyphenols, as effective antioxidants, protect our body against such diseases as cancers, diabetes, cardiovascular diseases and atherosclerosis. Recent *in vivo* and *in vitro* research has demonstrated that polyphenol compounds show beneficial effects on health by reducing the incidence of these diseases [1,2].

The medicinal properties of polyphenols connected with their biological activity of free radical scavenging were investigated by Rice-Evans *et al.* [3] and Iriti [4]. Polyphenols have been determined as some of the most essential non-nutrient components [3,4]. Bee pollen is a bee product characterised by biological activity due to its rich polyphenol composition. The powerful antioxidative properties of polyphenols result from the presence of double bonds, and the location of hydroxyl groups on the aromatic ring [5,6,7].

The ring structure of polyphenols determines their lipophilic properties, especially in the case of flavonoids. Hydrophobic antioxidants play a protective role for lipid membranes [8,9,10]. Polyphenols can scavenge reactive oxygen species (ROS) and inactivate organic radicals, and are able to complex metal ions which catalyse oxidation reactions [2,11,12,13,14,15,16,17,18,19].

Bee pollen possesses a powerful antioxidative activity owing to various compounds contained in it, which has been confirmed in many studies [2,7,12,13,14,16,17,18,19,20,21]. Furthermore, bee pollen is a source of hydrophilic antioxidants which protect against oxidative structure damage to the cytoplasm, inside cell organelles, and in the extracellular fluid. Among hydrophilic antioxidants, ascorbate (vitamin C) and phenolic acids can be found. They are hydrophilic to an extent and can also be effective antioxidants in an aqueous phase, e.g., caffeic acid [3].

Despite the fact that inhaled pollen may cause an allergic reaction, bee pollen in small amounts can be used for desensitization against hay fever. Resent research has indicated that bee pollen has an antiallergic activity because it protects mast cells from degranulation, and inhibits histamine release [22].

Bee pollen is not only a source of antioxidants with a wide range of activities, it also has a great nutrient value. Bee pollen contains carbohydrates, proteins and amino acids. It constitutes a rich source of exogenous amino acids with branched chains, *i.e.*, leucine, isoleucine, and valine. It also contains lipids—especially unsaturated fatty acids, vitamins and mineral substances. Owing to its nutrient and biotic properties, preparations made of bee pollen are used as dietary supplements, while bee pollen extracts can be used for enriching food in substances with pro-health effects, and for making nutraceuticals.

## 2. Bee Pollen

Bee pollen, called pollen load, is a bee product of plant origin, varying in its chemical composition. The composition of pollen depends on the flora present in various climate zones [23].

Over 250 biologically active substances of botanic origin have been isolated from bee pollen. Pollen, obtained by bees from the flowers of herbaceous plants and trees, constitutes a rich source of biologically active substances. Bees mix pollen with a small amount of saliva or nectar and, in the form of pollen loads, carry it to the hive in pollen baskets on their rear legs. Pollen in the form of pollen loads is obtained by means of pollen traps and, following drying, becomes raw material for pharmaceutical, cosmetic or nutritional use. Bee pollen is used in the production of dietary supplements in the form of tablets, capsules and granulates. Alcohol and aqueous extracts are also made from it.

Research by Nagai *et al.* [13] and Rzepecka-Stojko *et al.* [24] has demonstrated that enzymatic extraction with the use of pepsin is possible. In order to obtain the greatest amount of polyphenols, ethanol extraction of the sediment remaining after prior enzymatic extraction has been developed [24]. Extracts of bee pollen may be used to make functional food. In the search of the greatest amount of polyphenols, researchers develop various methods of pollen extraction [2,25].

### 2.1. Polyphenols

Polyphenols are components of flower bee pollen that determine its antioxidative activity [12,17,26]. Their content amounts to 3%–5% and may vary significantly depending on the origin of the raw material [27]. The profile of phenolic compounds in bee pollen—due to its specificity, and qualitative and quantitative stability—may serve as an important indicator of the quality of pollen loads [28]. According to their structure, polyphenol compounds in bee pollen can be differentiated into flavonoids and phenolic acids.

#### 2.1.1. Phenolic Acids

**Figure 1:**
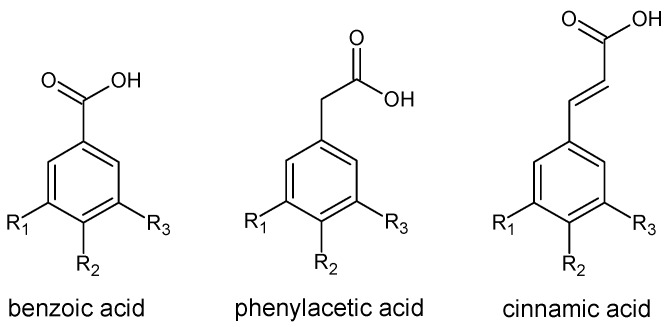
The basic structures of phenolic acids.

Phenolic acids are bioactive components of pollen. Their content in bee pollen amounts on average to 0.19%. They constitute a group of varied structures and properties. Their molecules contain an aromatic ring and a carboxyl group. Among them, we can differentiate benzoic acids, phenylacetic acids and cinnamic acids (Figure 1).

**Figure 2:**
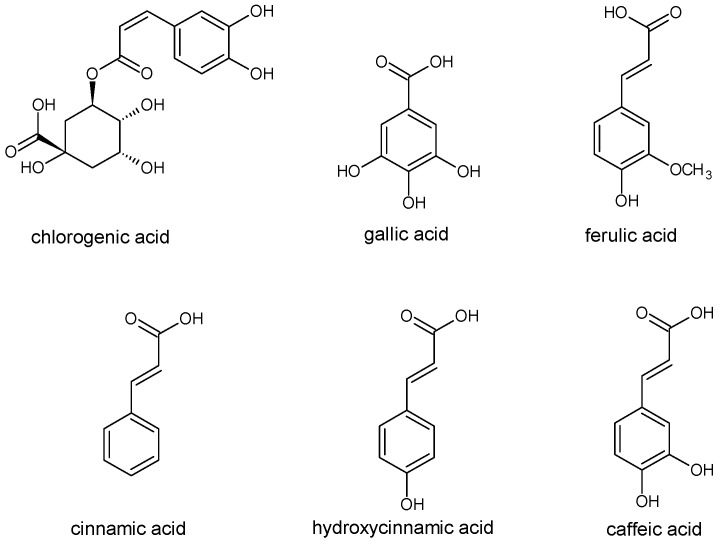
The structure of selected phenolic acids.

The derivatives of cinnamic and benzoic acids are of the greatest significance (Figure 2). Hydroxylated cinnamic acid derivatives are more effective antioxidants than benzoic acid derivatives. The antioxidative activity of phenolic acids is determined by the number of hydroxyl groups, the placement of functional groups, and any steric effects caused by them. Monohydroxy derivatives of benzoic acid have the best antioxidative properties in *meta*-hydroxylation, whereas, dihydroxy derivatives of benzoic acid are characterised by a high antioxidative activity in *ortho*- and *meta*-hydroxylation. The proximity of the COOH- group to *ortho*-diphenolic functional groups influences the accessibility of hydrogen in the *meta* position, which guarantees the greatest antioxidative effectiveness. Gallic acid (3,4,5-trihydroxybenzoic acid) has great antioxidant capacity, at the level of 3 mM Trolox equivalent antioxidant capacity (TEAC), owing to its three accessible hydroxyl groups. Attaching methyl groups to the 3-OH and 5-OH groups decreases its activity in comparison with trihydroxy derivatives. The addition of the third hydroxyl group to resorcinol (1,3-benzenediol) in the *meta* position decreases the antioxidant activity. Pyrogallol (1,2,3-trihydroxybenzene) can serve here as an example [3].

Cinnamic acid derivatives are more effective antioxidants than benzoic acid derivatives because of the introduction of an ethylene group between the phenyl ring and the carboxyl group, which increases the ability to release hydrogen. Monohydroxy derivatives of cinnamic acids are more accessible hydrogen donors than monohydroxy derivatives of phenylacetic acid. Additionally, the introduction of the second OH group in the *ortho* position as in caffeic acid, or in the *para* position, as in protocatechin acid, increases their antioxidative effects. Therefore, diphenols such as caffeic, chlorogenic and protocatechin acids demonstrate a greater ability to scavenge radicals than monophenols, corresponding to *para*-coumaric acid. The substitution of 3-OH group with methoxyl group in caffeic acid, as it is done in ferulic acid, results in an increase of antioxidative activity in the lipid phase [29,30].

The most common phenolic acids are chlorogenic, gallic, ferulic, cinnamic [16,31] and caffeic acids [32], as well as hydroxycinnamic, *ortho*-coumaric and *para*-coumaric acids [16,17,33]. In phenolic compounds present in pollen, the following phenylpropanoids [7] and derivatives of benzoic acid were determined: 3,4-dihydroxybenzoic acid, 4-hydroxybenzoic and vanillic acids [33,34], and 4-hydroxybenzoic acid ethyl ester [33].

#### 2.1.2. Flavonoids

**Figure 3:**
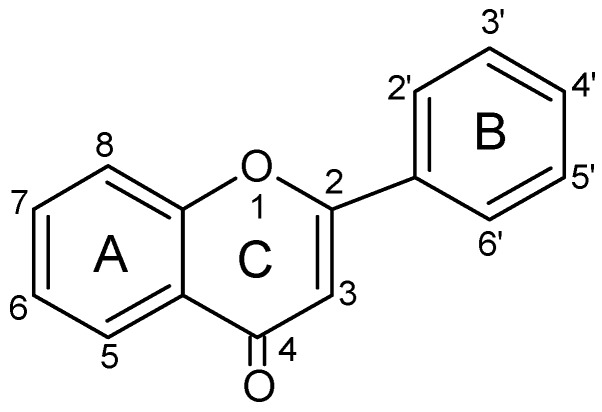
The basic structure of flavonoids.

Flavonoids constitute the most significant group of compounds among polyphenols present in bee pollen. The chemical structure of flavonoids is characterised by the presence of a diphenylpropan ring system (C6-C3-C6) with a benzo-γ-pyrone skeleton (Figure 3).

The presence of a double bond between C2 and C3 in the C ring in a flavonoid structure influences the flavonoid antioxidative properties. A carbonyl group at the C4 position enables the compounds to scavenge hydroxyl radicals. The presence of a OH group at the C3 position in the C ring allows the compounds to inhibit the peroxidation of lipids.

The ability to scavenge hydroxyl radical increases with the number of hydroxyl groups present in the B ring, especially at the positions 3′ and 4′. The presence of hydroxyl groups at the C5 and C7 positions in the A ring, C3′ and C4′ in the B ring, as well as C3 in the C ring enhances the inhibition of lipid peroxidation [3,4,35,36,37].

Seven groups of flavonoids are distinguished because of their chemical structure: flavons, flavonols, flavanons, flavanes, anthocyans, isoflavons, and chalcones. Flavonoids are present in pollen mainly in the form of glycosides, that is molecules with a sugar group, among which flavonol glycosides are present in greatest amounts [7,15,17,33]. The presence of a glycoside bond reduces antioxidative properties because of steric effects [37].

The level of free aglycones (flavonoids) is a better indicator of the quality of pollen loads than the free amino acids content [33]. We can distinguish flavonols, flavones, flavanones and isoflavones as the flavonoid components of bee pollen. The presence of particular flavonoids in pollen loads differs depending on plant species from which pollen comes [7,38].

**Figure 4:**
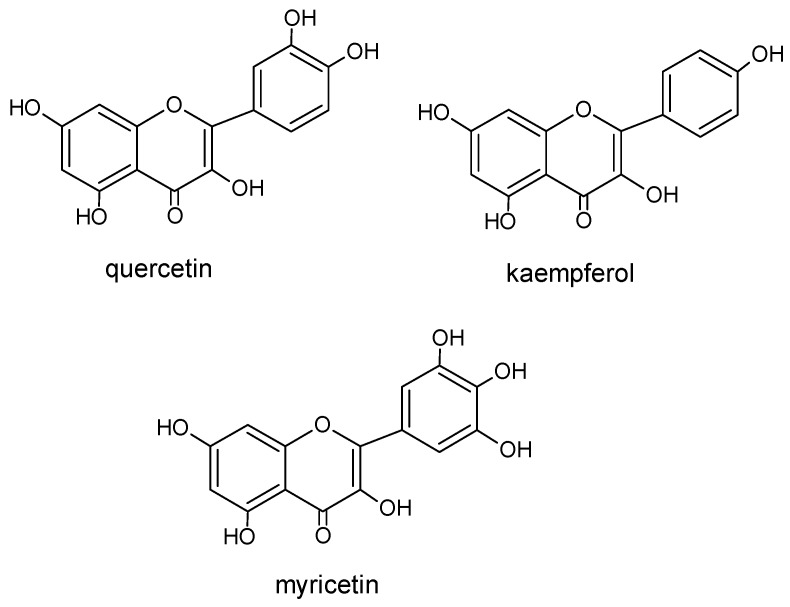
The structure of selected flavonoids.

During research on the chemical composition of pollen loads, various forms and types of flavonoids were discovered. The main flavonols of bee pollen are quercetin and kaempherol (Figure 4), as well as their glycosides [14,16,17,28,33,39].

Among quercetin glycosides, a common combination is rutin (rutoside), that is quercetin 3-*O*-rutoside [33,39]. Glucose and rhamnose disaccharide constitute its sugar part. The structure of quercetin (Figure 4) is responsible for its biological properties. In some authors’ opinion, the appropriate level of rutin content determines the biological and nutritional quality of pollen loads [33].

Other quercetin glycosides present in bee pollen are as follows: hyperoside, that is, quercetin 3-*O*-galactoside [39], quercetrin, that is, quercetin 3-*O*-rhamnoside [17,39], isoquercetrin, that is, quercetin 3-*O*-glucoside, as well as quercetin 3,7-*O*-diglucoside and quercetin-3-*O*-sophoroside [14,16,17,31]. Furthermore, among flavonoids in bee pollen, myricetin (Figure 4) and its glycosides [33], as well as 8-methoxyherbacetin, tricetin [38,40], isoramnetin and galangin [17,32,38,39] have been identified. Less common flavonoids are specific glycosides characteristic for particular plants, which are the source of 8-*O*-methylherbacetin-3-*O*-glucoside, characteristic of pollen load collected from *Raphanus raphanistrum* L. plants [7,41] and 8-methoxykaempherol 3-neohesperidoside, 8-methoxy kaempherol 3-glucoside, and kaempherol 3-neohesperidoside from the common hawthorn *Crataegus monogyna* Jacq. [42].

Flavones present in bee pollen are as follows: apigenin glycosides—vitexine, that is, apigenin C-glycoside [17,28,39] and vitexin *O*-rhamnoside, luteolin glycosides [17,28] and chrysin [32].

Flavanones identified in bee pollen are naringenin [38] and pinocembrin [32]. Among isoflavones, genistein glycosides [17,28] and selagin [38] have been detected in pollen. Genistein has an isoflavone structure and is considered to be a phytoestrogen because of its ability to bind with oestrogen receptors. This property is closely related to the hypolipemic and anticancerogenic properties of bee pollen [8,43,44,45].

Other flavonoids in bee pollen are leucoanthocyanidins and catechins. The average content of the former ones amounts to 0.27%. Catechins are the least numerous group of flavonoids in bee pollen and are, on average, present in an amount of 0.09% [31]. The total flavonoid content, both of free flavonoids and in glycoside-bound forms, varies between 0.25% and 1.4% [31]. Bogdanov gives the range of 0.04%–3% (40–3000 mg/100 g) as the norm [46]. Table 1 lists the basic polyphenolic compounds of bee pollen.

**Table 1:** Main polyphenolic compounds of bee pollen.

MAIN POLYPHENOLIC COMPOUNDS OF BEE POLLEN			
Bee Pollen Compound and the Structures of Major Classes		Free Hydroxyl Groups Position [3,36]	TEAC ^a^ (mM) [3]
1. PHENOLIC ACIDS			
*HYDROXYBENZOIC ACIDS:*			
Gallic acid	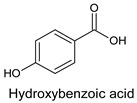	3,4,5	3,0
Protocatechuic acid		3,4	1,2
*HYDROXYCINNAMIC ACIDS:*			
Caffeic acid	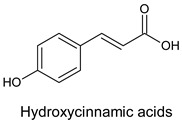	3,4	1,3
Ferulic acid		4	1,9
Chlorogenic acid		3,4	1,3
*para*-Coumaric acid		4	2,2
*ortho*-Coumaric acid		2	1,0
2. FLAVONOIDS			
*FLAVONES:*			
Luteolin	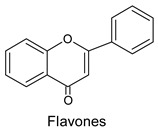	5,7,3′,4′	2,1
Apigenin		5,7,4′	1,5
Chrysin		5,7	1,4
*FLAVONOLS:*			
Quercetin	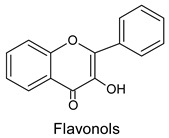	3,5,7,3′,4′	4,7
Rutin (Q 3-o-rutoside)		5,7,3′,4′	2,4
Kaempherol		3,5,7,4′	1,3
Myricetin		3,5,7,3′,4′,5′	3,1
Galangin		3,5,7	N/D
*FLAVANONES:*			
Naringenin	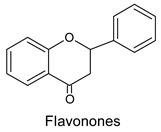	5,7,4′	1,5
Pinocembrin		5,7	N/D
*ISOFLAVONES:*			
Genistein	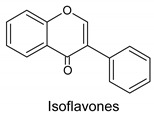	5,7,4′	N/D

^a^ Trolox equivalent antioxidant capacity.

## 3. Absorption and Metabolism of Phenolic Acids and Flavonoids

The efficiency of polyphenols absorption depends on their physicochemical properties: the size of the molecule, the presence of functional groups, spherical configuration, lipophilicity, and solubility. The forms soluble in water are better absorbed from the gastrointestinal tract than lipophilic compounds. Polyphenols in the chemically bound forms are subjected to the activity of bowel microflora enzymes and absorbed in the final section of the bowel [11].

The research by Konishi *et al.* [47] has demonstrated that the absorption of gallic acid is significantly lower than that of caffeic acid. The absorption of hydroxyaromatic acids, benzoates, phenylacetates and hydroxycinnamates requires breaking down the polyphenol in the gastrointestinal tract to a lower molecular form.

The absorption of both flavonoids and phenolic acids depends on their structure. Quercetin and genistein aglycones are directly absorbed by the intestinal mucosa, and correspondingly metabolized to caffeic acid. Donovan *et al.* [48] estimated that the absorption of quercetin aglycones in rat small intestine amounts to 67%.

However, in natural products only small amounts of aglycones are present. Flavonoid glycosides are less absorbed due to their hydrophilic nature. Isoflavones are an exception here, since no difference has been determined between aglycones and glycosides. Hydrolysis of flavonoid glycosides can effectively take place in the whole gastrointestinal tract, including the oral cavity. Hollman *et al.* [49] demonstrated that hydrophilic quercetin glycosides can be transported to the small intestine by intestinal Na^+^-dependent glucose transporter 1 (SGLT1). Flavonoid glycosides can penetrate enterocytes where they are hydrolised by a broad-specific-β-glucosidase enzyme (BSPβG). In addition, lactase phlorizin hydrolase (LPH) located in the brush border of the mammalian small intestine may participate in flavonoid glycoside hydrolysis. It is known that the sugar moiety of glycosides is a major determinant of their absorption, for example the absorption of pure quercetin-3-β-rutinoside amounts to 20% compared to pure quercetin-4′-β-glucoside. Moreover, food may influence the absorption of these compounds—for example, milk decreases the absorption of flavonols [49,50].

Polyphenols ingested with food affect the gastrointestinal tract. Polyphenols with a pyran ring and hydroxyl functional groups increase the solubility of chyme. Quercetin activates fermentation processes, playing a protective role against toxins in food [51]. The metabolism of flavonoids occurs along three paths: oxidation, glucuronisation and sulphonation. Oxidation of flavonoids takes place with the participation of cytochrome P450 enzymes. Many studies have demonstrated the inhibiting influence of flavonoids on CYPs, especially CYP1A1/1A2. The enzymes participate in the oxidation of galangin in the following order: CYP2C9, and then CYP1A2 and CYP1A1. In kaempferide oxidation, CYP1A2 takes part first, and then CYP2C9 and CYP1A1 play a dominant role. Research has shown that CYP1A2 and CYP2E1 participate in the oxidation of numerous isoflavones. Coupled metabolism competes with flavonoid oxidation because after the absorption of polyphenols into enterocytes, the compounds undergo glucuronisation. The generation of glucuronic acid conjugates with quercetin, luteolin, chrysin and diosmetin occurs with the participation of UDP-glucuronosulphotransferase (UGT). The attachment of glucuronic acid to a polyphenol molecule depends on the number, as well as on the location of hydroxyl groups. Furthermore, methylation may also take place in enterocytes. In the case of quercetin, the process takes place at the 3′ and 4′ positions of the aromatic ring. Depending on the position where the reactions take place, the antioxidative properties and the biological activity of the compounds may vary [48,52].

Quercetin has a high antioxidative potential, while its glucuronides and sulphates are only partially as potent. In studies on human cells, covalent binding of oxidised quercetin to DNA and cellular protein has been determined. The interaction with proteins may influence flavonoid biological activity, but further studies are required to confirm that. Polyphenols bind to blood albumins in the small and large intestines. During the transport to the liver, glucuronide bonds are dissociated and then metabolised to glucuronide and sulphate conjugates, therefore they become more water-soluble. This process prevents the accumulation of polyphenols and their metabolites in the liver. In the liver, catechol-*O*-methyltransferase is responsible for methylation reactions of flavonoids, while sulphotransferase accounts for their reactions with sulphates [48,52,53].

Catechol-like flavonoid structures are susceptible to *ortho*-methylation by soluble catechol-*O*-methyltransferase (COMT). Olthof *et al.* [54] state that binding with glucuronic and sulphuric acids decreases antioxidative properties of phenolic acids. Quercetin-4′-glucuronide demonstrates a very low antioxidant activity, quercetin-3-glucuronide is quite potent, but less than quercetin [55]. In the case of quercetin-4′-glucoside and quercetin-3-glucoside the situation is similar. From the liver, polyphenols are transported to the blood, where they stay for a time interval different for particular compound. Quercetin is present there for over a dozen hours, and then it is eliminated. Polyphenols are excreted mainly with urine, but they can also be excreted with faeces, and according to King *et al.* [56], 21% of isoflavones ingested with food are excreted with faeces.

Flavonoids are effectively metabolised by the cells of the gastrointestinal tract, and then excreted with faeces in the form of glucuronides and sulphates. The main product of quercetin excretion is carbon dioxide measured by trapping exhaled air, which indicates that bacteria from the lower part of the intestines constitute one of the steps of flavonoid elimination. Polyphenols, which have not been excreted from the body, are transported back from the blood to tissues and may have biological effects [57]. Fiorani *et al.* [58] state that quercetin, myricetin and genistein affect red blood cells metabolism. They are accumulated in red blood cells and perform antioxidative functions. In normal diet, flavonoid concentration in the blood serum is lower than 1 µM [50].

## 4. Properties of Bee Pollen

Around 70% of substances in bee pollen are biologically active. Therefore, this natural bee product has multidirectional effects. It demonstrates nutritive, antioxidative, cardioprotective, hepatoprotective, anti-inflammatory, antibacterial, anticancerogenic, immunostimulant, and antianaemic effects. The basic properties of bee pollen are shown in Table 2.

**Table 2:** Properties of bee pollen.

Main Effect	Mechanism of the Biological Activity
Nutritive	Source of proteins (23.9% ^a^; vital role), exogenous amino acids (8.6% ^a^; vital role, malnutrition), carbohydrates (13%–55% ^a^), lipids (0.3%–20% ^a^; hypolipidemic) including unsaturated fatty acids (2.7% ^a^; anti-inflammatory, anticancerogenic), phospholipids (1.5% ^a^; cardioprotective, anti-inflammatory), phytosterols (1.1% ^a^; antiartherosclerotic) vitamins (0.7% ^a^), bioelements (1.6% ^a^) [22,31,59,60]
Antioxidative	Scavenging hydroxyl radicals; complexing metals [7,12,13,14,17,18,19,20,21]
Cardioprotective	Inhibition of ACE activity; inhibition of blood platelets aggregation [61,62,63]
Hepatoprotective	Reduction of lipofuscin; detoxifying activity in industrial poisoning [64]
Anti-inflammatory	Inhibition of COX-2; inhibition of NO production [25,65,66,67,68,69,70,71]
Antibacterial	Disruption of bacteria metabolism, especially in: *Staphyllococcus aureus*, *Staphylococcus epidermidis*, *Bacillus cereus*, *Bacillus subtilis*, *Pseudomonas aeruginosa*, *Salmonella enterica*, *Listeria monocytogenes*, *Escherichia coli* [72,73,74]
Anticarcinogenic	*Brassica camperstris* L. bee pollen extract increases the activity of caspase-3 enzyme, and decreases the expression of anti-apoptic proteins Bcl-2; *Cistus incanus* L. and *Salix alba* L. bee pollens inhibit 17β-estradiol activity [75,76]
Antianaemic	Decrease of the number of blood platelets; increase in haemoglobin level [77,78]
Effects on bone tissue	*Cystus ladaniferus* L. bee pollen inhibits the resorption of the femur and formation of osteoclastic cells, and increases the level of alkaline phosphatase [79,80,81]

^a^ average content in raw bee pollen.

### 4.1. Nutritive Properties

Considering its nutritional value, bee pollen is primarily a source of nutritious protein [12,82,83]. Its protein content amounts on average to 23.9% of the product dry mass [31]. Despite the fact that the protein content in pollens of different botanical origin varies, the average protein content in pollen is similar, irrespective of the parts of the globe a particular pollen comes from. According to Szczęsna and Rybak-Chmielewska [84], the protein content in Polish pollen amounts to 20.7%, whereas, it is 17% in Spanish pollen, 20.7% in Korean pollen and 23.7% in a Chinese one. Almeida-Muradian [26] states that in Brazilian pollen, the protein content is 21.4%. Some authors refer to pollen as the amino acid essence, as the content of these compounds is many times higher than in high protein animal products [83,85,86,87]. Pollen contains all exogenous amino acids, as well as those relatively exogenous, such as arginine and histidine. Pollen is also a rich source of carbohydrates and lipids, including unsaturated fatty acids, bioelements, vitamins, especially carotenoids as well as polyphenol compounds, mainly flavonoids [2,85,88,89]. Carbohydrates are pollen components which are present in it in the greatest amounts. They comprise both reducing sugars, such as fructose, glucose and maltose, but also non-reducing ones, such as sucrose. Carbohydrates constitute between 13% and 55% of pollen. Portuguese pollen of pine, corn and bullrush contains 13.92%, 36.59% and 31.93% of carbohydrates, respectively [59]. According to Szczęsna’s study [83], Chinese pollen contains 26.9% of carbohydrates and a Korean one contains 48.8%. Lipids in bee pollen constitute between 0.3% and 20% and comprise unsaturated fatty acids, such as palmitoleic acid, oleic acid, α-linolenic acid and arachidonic acid, as well as saturated fatty acids. Among the saturated fatty acids identified in bee pollen, there are caproic, caprylic, lauric, myristic, palmitic and stearic acids. The ratio of unsaturated acids to saturated ones is 2.67. Bee pollen contains on average 2.7% of essential unsaturated fatty acids, which is reflected in its special nutritional value [31]. Phospholipids are also present in the pollen: phosphatidylcholine, phosphatidylethanolamine, phosphatidylinositol; and phytosterols: β-sitosterol, campesterol. Owing to the presence of phospholipids, which are lipotropic agents, bee pollen plays an important role in metabolic transformations, while its phytosterol content gives it an oestrogen function in both, human and animal body [60].

Apart from nutritional components such as proteins, carbohydrates and lipids, bee pollen contains numerous bioelements: sodium, potassium, magnesium, calcium, phosphorus, as well as manganese, iron, cobalt, nickel, copper and zinc. Bee pollen is not only a valuable source of bioelements supplementing their insufficiency in human body, but also a rich source of vitamins. Their content in pollen amounts to 0.7%. Bee pollen contains significant amounts of group B vitamins, biotin, and ascorbic acid. Bee pollen collected from willow, pear and apple trees, as well as from dandelion, is characterised by the highest content of ascorbic acid [31]. Vitamins soluble in lipids constitute only 0.1%. β-carotene is present in the greatest amount, as well as other carotenoids, vitamin E and calciferol [22].

Polyphenols, namely, phenolic acids and flavonoids are responsible for a wide range of bee pollen biological activities. Therefore, bee pollen with propolis, honey and beebread is a component of dietetic preparations enhancing health [87]. Because of its content, bee pollen may replace many other health enhancing products [90].

### 4.2. Antioxidant Activity

Bee pollen is characterised by high antioxidative potential, which determines its biological activity [2,7]. It is a known fact that many diseases are caused by the negative effects of excessive amounts of reactive oxygen species (ROS) [10,15,20,43]. According to Bartosz [9], there are three essential types of relationships in which the changes in the ROS level are a cause, mediator or consequence of disease processes. Among the diseases related to oxidative stress, we can enumerate: inflammations, reperfusion following ischaemia, rheumatoid arthritis, atherosclerosis, hypertension, diabetes, diseases of the central nervous system, gastrointestinal tract inflammations and ulcerations, tumours, AIDS, auto-immunological diseases, cystic fibrosis, renal diseases and accelerated-ageing syndromes [9,37].

Owing to its high content of various polyphenolic compounds, bee pollen demonstrates powerful antioxidative effects, which have been confirmed by many tests [7,12,13,14,16,17,18,19,20,21]. Among antioxidants present in bee pollen, low molecular weight compounds are the most significant. Ascorbate (vitamin C) and polyphenolic compounds belong to the hydrophilic antioxidants. Whereas, tocopherols (vitamin E) and carotenoids belong to the hydrophobic antioxidants. As a water phase antioxidant, vitamin C scavenges hydroxyl radicals. It contributes to retaining the proper level of NO in oxidative stress, so that the compound can demonstrate relaxing effects on arterial smooth muscle [91,92,93].

The antioxidative effects of vitamin E consists in reacting with ROS, organic free radicals and its ability to terminate lipid peroxidation. It is the most significant hydrophobic antioxidant. In turn, carotenoids have the ability to quench singlet oxygen, reduce organic free radicals and inhibit lipid peroxidation, especially in low density lipoprotein (LDL). Therefore, they are thought to prevent atherosclerosis [8,10,15,43,44]. The most significant antioxidants in bee pollen are polyphenols, especially flavonoids. Apart from their ability to scavenge ROS and inactivate organic radicals, they can complex metals which catalyse oxidation reactions [2,11,12,13,14,16,17,19].

### 4.3. Cardioprotective Effects

The beneficial effects of bee pollen on the cardiovascular system are connected with the presence of essential unsaturated fatty acids, vitamin E, phytosterols, phospholipids and flavonoids [2,12,17,20,65,93,94].

A huge effect of bee pollen hydrolysates on an enzyme converting angiotensin I to angiotensin II (ACE), has been demonstrated. The results indicate a high antioxidative potential of bee pollen—manifesting itself by the inhibition of ACE activity—which results in hypotensive effects [61]. Other authors indicate the possibility of antiatherosclerotic effects of bee pollen [95,96].

In the available literature, there are many reports concerning the properties of flower pollen of various botanic origins. The conducted tests describe hypolipemic effects and blood platelet aggregation inhibition [62,63].

### 4.4. Hepatoprotective Effects

Results of many tests conducted on animals have confirmed the hepatoprotective and detoxifying activities of bee pollen.

It has been determined that bee pollen extracts influence the process of carbaryl intoxication in rats. Pollen water extracts decreased oxidative stress markers, such as: MDA, CAT, SOD, GSH-Px and improved biochemical parameters, such as total protein albumins, glucose, triglycerides, total bilirubin, creatinine, urea, magnesium, sodium, potassium, chloride and hepatic enzymes GGT, LDH, AST, ALT and ALP, which were disturbed as a result of carbaryl activity [21]. In old rat livers, bee pollen normalised the content of malondialdehyde and sulphydryl groups, as well as the level of proteins and urea [97].

The effect of bee pollen was also tested on intracellular lipofuscin in mice. Lipofuscin is an intracellular syndrome of ageing process. It is composed of oxidised and crosslinked lipids and proteins. The pigment is produced in mature cells. It accumulates with age and may occupy as much as one half the volume of a cell. It has been demonstrated that bee pollen induced the reduction of lipofuscin in myocardium, liver, brain and adrenal glands. This activity may be connected with the antioxidative properties [64].

Furthermore, the effect of bee pollen on prolonged exposition to ionising radiation in the dose of 0.25 Gy and cadmium chloride in rats was estimated. These factors lead to a decrease in the level of intracellular potassium in the brain. Administration of oil with β-carotene and bee pollen limited the effect of radiation, but did not eliminate toxicity [98]. The detoxifying activity of bee pollen in industrial poisoning, and ethanol poisoning has also been emphasized [99,100].

### 4.5. Anti-Inflammatory Properties

Many studies concerning the properties of bee pollen indicate its anti-inflammatory properties, resulting mainly from the content of phenolic acids and flavonoids, as well as phytosterols [65,66,94].

Tests conducted on rats have demonstrated the powerful anti-inflammatory properties of bee pollen ethanol extracts in response to the inflammatory state caused by carrageenan exposure. Moreover, the mechanism of the anti-inflammatory activity of bee pollen extracts has been determined. Ethanol extract inhibited the inducible COX-2 isoform more effectively than the constitutive COX-1, which directly translates into safety and selectiveness of its activity. Furthermore, the inhibition of NO production has been demonstrated by inducible nitric oxide synthase (i-NOS), the activity of which has been determined in the inflammatory response [25].

The anti-inflammatory properties of bee pollen have been confirmed in clinical tests of benign prostatic hyperplasia (BPH). The tests, involving a double-blind test, assessed the effectiveness and safety of bee pollen supplementation for the period of 12 weeks. The tested patients were divided into 3 groups: 1—placebo, 2—receiving low-dose supplementation of 160 mg of extract/day, 3—receiving high-dose supplementation of 320 mg of extract/day. After 12 weeks a significant increase in urine pressure in groups supplemented with bee pollen was determined. In the placebo group, however, a decrease in the pressure was noted. Based on research, it was also stated that a high dose of bee pollen extract alleviated the symptoms of benign prostatic hyperplasia, having a positive effect on such parameters as prostate volume, urine volume and urine flow [101].

Similar studies have been conducted on dogs, which were administered flower bee pollen in the dose of 5–10 g/kg of body mass, orally for 2 months. A positive therapeutic effect was obtained in BPH, manifested by the improvement in morphological parameters [102]. Furthermore, the literature offers many reports on the anti-inflammatory and therapeutic effects of various types of flower bee pollen extracts in BPH [67,68,69,70,71].

### 4.6. Antibacterial Effects

Ethanol extracts of bee pollen demonstrate quite powerful antibiotic properties against pathogenic Gram+ and Gram− bacteria, as well as to pathogenic fungi. This results from the presence of flavonoids and phenolic acids in bee pollen.

Flavonoid effects on bacteria are connected with the disruption of their metabolism. The mechanism consists in forming complexes with bacterial cell walls by surface-exposed adhesin and polypeptides, and/or cell membrane enzymes, which leads to the disruption of cell wall integrity, blocking ion channels, and inhibiting electron flow in the electron transport chain that determines adenosine triphosphate (ATP) synthesis, by scavenging electrons [8].

The antibiotic activity of flavonoids isolated from bee pollen against *Pseudomonas aeruginosa* has been demonstrated [71]. Hydrophobic components of bee pollen were tested as to their antibacterial activity, and their effects against *Streptococcus viridans* have been demonstrated [103]. Furthermore, substances present in bee pollen which display antibacterial activity against *Staphyloccocus aureus* are similar to those found in propolis and honey [104]. In studies with the use of Brazilian bee pollen ethanol extracts, the antibacterial activity against *Staphylococcus aureus*, *Bacillus cereus*, *Bacillus subtilis*, *Pseudomonas aeruginosa* and *Klebsiella* sp. has been determined [105].

The antibacterial activity of bee pollen against *Staphyloccocus aureus* and *Staphyloccocus epidermidis* was also substantiated by Baltrusaitye [72].

Bee pollen antibacterial activity has been assessed against many microorganisms, such as *Pseudomonas aeruginosa*, *Listeria monocytogenes*, *Staphylococcus aureus*, *Salmonella enterica* and *Escherichia coli*. All tested bacterial strains were characterised by sensitivity to bee pollen extracts as early as in the first 24 h of incubation. *Pseudomonas aeruginosa* demonstrated the greatest sensitivity [73].

Methanol and ethanol extracts of bee pollen were characterised by similar antibacterial activity against the aforementioned Gram+ and Gram – bacteria. Additionally, they displayed antifungal properties against *Aspergillus fumigatus, Aspergillus niger, Aspergillus flavus* and the yeasts: *Candida albicans*, *Candida glabrata*, *Candida krusi* and *Rhodotorula mucilaginosa* [106]. Antifungal and antibacterial activity was also established in the case of Greek bee pollen. It has been ascertained that the antifungal and antibacterial activity might be caused by high quercetin and kaempferol content in the tested extracts of bee pollen [14]. The antibacterial activity of Turkish bee pollen has been proven against over a dozen plant bacterial pathogens. Therefore, pollen extracts may be used to protect plants instead of such substances as copper compounds or pesticides, whose use has been limited [74].

### 4.7. Anticarcinogenic Properties

Thanks to phenolic components, as well as non-phenolic antioxidants, bee pollen extracts demonstrate cytotoxic properties against many tumours. The studies on the effects of *Brassica campestris* L. bee pollen extracts on the vitality of the cells of human prostate cancer have demonstrated that the sterol fraction of a chloroform extract significantly increases the activity of caspase-3 enzyme, and causes a decrease in the expression of anti-apoptotic proteins Bcl-2. This results in cytotoxicity towards the cells of human androgen-independent prostate cancer PC-3, leading to their apoptosis. The obtained results indicate that the steroid fraction of *Brassica campestris* L. bee pollen chloroform extract may be a promising candidate for advanced prostate cancer treatment [75].

Furthermore, anti-estrogenic and antigenotoxic activities of bee pollen extracts from cistus (*Cistus incanus* L.) and white willow (*Salix alba* L.) have been demonstrated. The extracts from the tested materials were effective inhibitors of natural 17β-estradiol activity, and caused a decrease in the extent of the damage to human lymphocytes subjected to anticancer drugs such as: bleomycin, mitomycin c, and vincristine. The level of activity depended on the cumulative polyphenol content. This is significant in oestrogen-dependent cancers, for example, breast cancer in women, and may find its application as a protector against the mutagenic effects of anticancer drugs [76].

The anti-oestrogenic activity of bee pollen extracts has been confirmed in tests conducted *in vivo* and *in vitro*. The results indicate the possibility of using the pollen to decrease the risk of the disease in the case of hormone-dependent breast, uterus and prostate cancers, as well as to improve the functioning of prostate in elderly men [32].

In tests *in vitro*, it has been demonstrated that bee pollen is characterised by angiostatic effects due to its influence on processes regulating endothelial cells proliferation and migration by blocking the expression of VEGF. Thus, it may be used as a potential therapeutic factor in the course of proangiogenic diseases [107].

Furthermore, based on research, it has been established that bee bread ethanol extracts demonstrate cytotoxic activity against glioma cell lines. The significant role of linolenic acid and its stereoisomer is indicated in the course of glioma [95].

### 4.8. Immunostimulatory Activity

It has been demonstrated that polysaccharide fractions obtained from bee pollen stimulate immunological activity through an increase in macrophage phagocytic index, mainly the increase in the number of phagocytes, and they have beneficial effects on splenocyte and NK lymphocyte proliferation [108].

In *in vitro* and *in vivo* tests conducted on mice, an inhibiting effect of bee pollen extracts has been determined on the activation of mast cells induced by immunoglobulins E (IgE). Bee pollen extracts, both *in vitro* and *in vivo* conditions, significantly decrease mast cell degranulation as a result of a decrease in the level of tyrosine phosphorylation. Furthermore, tests *in vitro* confirmed the decrease in the production of tumour necrosis factor TNF-α. The results indicated that antiallergic effects of bee pollen extracts play a significant role not only in the initial, but also in the final phase of allergic reactions [109].

### 4.9. Antianaemic Effects

Bee pollen may significantly decrease the negative effects of iron deficiency, thus demonstrating antianaemic effects. Research on the influence of bee pollen and propolis on iron, calcium phosphorus and magnesium metabolism in rats with nutritional iron deficiency, treated as an experimental model of anaemia, demonstrated that bee pollen supplementation results in a decrease of the number of blood platelets, and an increase in haemoglobin level. Furthermore, weight gain was observed, as well as beneficial effects on magnesium, calcium and phosphorus metabolism. It was determined that the tested bee products alleviate to a great extent the negative consequences of iron deficiency, exerting restorative influence and improving the absorption and utilisation of nutritional iron [77].

The beneficial influence of bee pollen in the case of haemolytic anaemia in mice and rats has also been confirmed. It was determined that bee pollen caused haematopoietic system stimulation and reduced the level of white blood cells in the animals [78].

### 4.10. Effects on Osseous Tissue

Hamanoto *et al.*’s [79] research indicates that bee pollen exerts have a positive influence on osseous tissue. *Cystus ladaniferus* L. bee pollen aqueous extracts inhibit the resorption of the femur in rats and hinder the formation of osteoclastic cells in mice. It has been also determined that bee pollen aqueous extracts significantly increase the level of alkaline phosphatase, an enzyme which participates in bone mineralisation.

The same group of scientists has confirmed in both, *in vitro* and *in vivo* tests that oral administration of bee pollen aqueous extracts significantly increases calcium and alkaline phosphatase content enhancing anabolic effects in the growth zone and the fully developed part of rat femur [80].

Similar tests were carried out on a diabetic rat population, demonstrating the protective effects of bee pollen aqueous extracts in the case of bone loss in the course of diabetic osteoporosis, and causing a partial decrease in the levels of glucose and triglycerides in blood serum [81].

Tests *in vitro* demonstrated the anabolic effects of bee pollen extracts on the osteoblastic cells of MC3T3-E1 line, confirming that bee pollen extracts stimulate osteoblastic bone formation [110].

## 5. Conclusions

As a natural bee product of high antioxidative potential, bee pollen may effectively enhance protective mechanisms against reactive oxygen species, which are involved in a wide range of negative effects on human organisms. When an imbalance between generated ROS and available antioxidants occurs, oxidative damage will spread via free radical generation in human body. Bee pollen fulfils a preventive function against the development of many lifestyle diseases. It may also support pharmacological treatment. By learning about the therapeutic properties of bee pollen and the mechanism of its active components action, its beneficial influence on human health can be embraced. Precise knowledge about the metabolism of phenolic acids and flavonoids as well as the concentration of their metabolites in particular cells and tissues allows for a wider use of bee pollen, especially its standardised extracts in medical treatment.
