# Correction: Rzepecka-Stojko, A., *et al*. Polyphenols from Bee Pollen: Structure, Absorption, Metabolism and Biological Activity. *Molecules* 2015, *20*, 21732–21749

**DOI:** 10.3390/molecules21020159

**Published:** 2016-01-28

**Authors:** Anna Rzepecka-Stojko, Jerzy Stojko, Anna Kurek-Górecka, Michał Górecki, Agata Kabała-Dzik, Robert Kubina, Aleksandra Moździerz, Ewa Buszman

**Affiliations:** 1 Department of Pharmaceutical Chemistry, School of Pharmacy with the Division of Laboratory Medicine, Medical University of Silesia, Jagiellońska 4, Sosnowiec 41-200, Poland; ebuszman@sum.edu.pl; 2 Department of Hygiene, Bioanalysis and Environmental Studies, School of Pharmacy with the Division of Laboratory Medicine, Medical University of Silesia, Kasztanowa 3A, Sosnowiec 41-200, Poland; jstojko@sum.edu.pl (J.S.); amozdzierz@sum.edu.pl (A.M.); 3 Silesian Medical College in Katowice, Mickiewicza 29, Katowice 40-085, Poland; anna.kurek_gorecka@swsm.pl; 4 Department of Drug Technology, School of Pharmacy with the Division of Laboratory Medicine, Medical University of Silesia, Jedności 8, Sosnowiec 41-200, Poland; mgorecki@sum.edu.pl; 5 Department of Pathology, School of Pharmacy with the Division of Laboratory Medicine in Sosnowiec, Medical University of Silesia in Katowice, Ostrogórska 30, 41-200 Sosnowiec, Poland; adzik@sum.edu.pl (A.K.-D.); rkubina@sum.edu.pl (R.K.)

The authors wish to make the following corrections to this paper:

**Author Contributions:** Anna Rzepecka-Stojko designed the review; coordinated the writing and wrote Section 2, Section 2.1, Section 2.1.2, Section 4, Section 4.1, Section 4.2 and collaborated in the creation of Figures 3–5 and Table 1 and 2; Jerzy Stojko conceived the review, supervised the writing of all sections and wrote Abstract and Section 1, Section 4.8, Section 4.9, Section 4.10; Anna Kurek-Górecka wrote Section 2.1.1 and Section 3; Michał Górecki created Table 2; Agata Kabała-Dzik wrote Section 4.5 and Section 4.6; Robert Kubina wrote Section 1 and section 4.7 and collaborated in the creation of Table 1 and 2; Aleksandra Moździerz wrote Section 4.3 and 4.4; Ewa Bszman: Supervised the writing of all sections and wrote Conclusion. All authors were involved in the editing process. All authors discussed and approved the final version.

Should be corrected as:

**Author Contributions**: Anna Rzepecka-Stojko designed the review; coordinated and participated in the writing of all sections and wrote Sections 2, 2.1, 2.1.2, 4, 4.1 and 4.2 and collaborated in the creation of Figures 3 and 4 and Tables 1 and 2; Jerzy Stojko conceived the review, supervised the writing of all sections and wrote the Abstract and Sections 1, 4.8, 4.9 and 4.10; Anna Kurek-Górecka wrote Sections 2.1.1 and 3; Michał Górecki created Table 2; Agata Kabała-Dzik wrote Sections 4.5 and 4.6; Robert Kubina wrote Sections 1 and 4.7 and collaborated in the creation of Tables 1 and 2; Aleksandra Moździerz wrote Sections 4.3 and 4.4; Ewa Bszman supervised the writing of all sections and wrote the Conclusions. All authors were involved in the editing process. All authors discussed and approved the final version.

The authors would like to apologize for any inconvenience caused to the readers by these changes.

